# YELLOW RoUTIne prospective cohort study protocol: insight in the dynamics of bacteria in the elderly bladder

**DOI:** 10.1186/s12879-024-09727-w

**Published:** 2024-08-30

**Authors:** Ruo Chen Wang, Laura W. Van Buul, Suzanne E. Geerlings, Sabine C. De Greeff, Anja Haenen, Kati Halonen, Daan W. Notermans, E. Ascelijn Reuland, Martin Smalbrugge, Jos W. R. Twisk, Caroline Schneeberger

**Affiliations:** 1 Department of Medicine for Older People, Amsterdam UMC Location Vrije Universiteit, Amsterdam, The Netherlands; 2 Amsterdam Public Health Research Institute, Amsterdam, The Netherlands; 3 Department of Internal Medicine, Infectious Diseases Division, Amsterdam UMC Location University of Amsterdam, Amsterdam, The Netherlands; 4 Amsterdam Institute for Immunology and Infectious Diseases, Amsterdam, The Netherlands; 5 Centre for Infectious Disease Research, Epidemiology and Surveillance, National Institute for Public Health and Environment, Bilthoven, The Netherlands; 6 Centre for Infectious Disease Research, Diagnostics and Laboratory Surveillance, National Institute for Public Health and Environment, Bilthoven, The Netherlands; 7 Infectious Diseases in Primary Care, Nivel, Utrecht, The Netherlands; 8 Department of Medical Microbiology, Central Bacteriology and Serology Laboratory, Tergooi Medical Centre, Hilversum, The Netherlands; 9 Department of Epidemiology and Data Science, Amsterdam UMC Location Vrije Universiteit, Amsterdam, The Netherlands

**Keywords:** Older adults, Bacteriuria, Urinary tract infection, Nursing homes, Antibiotic stewardship

## Abstract

**Background:**

Asymptomatic bacteriuria (ASB) – the presence of bacteria in urine without urinary tract infection (UTI) related signs & symptoms (S&S) – is common in the elderly bladder and is not considered pathogenic for UTI. We hypothesise that colonisation with non-uropathogenic bacteria could protect the bladder from invasion of more harmful bacteria. The exact role and dynamics of bacteriuria in the relation to the development of a UTI is still unknown. We aim to provide insight into the course of bacteriuria in the elderly bladder and its relation to UTI in frail older adults.

**Methods and analysis:**

A prospective observational cohort study is being conducted in Dutch nursing homes (NHs) between February 2024 and December 2025. Urine samples and case report forms (CRF) on UTI-related S&S will be collected from each consenting NH resident every 3 months for a follow-up period of 18 months. Whenever a UTI-suspicion occurs in between the 3 monthly time points, additional data and a urine sample will be collected. Urine samples undergo several urinalyses (e.g. dipstick and bacterial culture). Additional molecular analysis will be conducted on a selection of cultured *Escherichia coli* (*E. coli*) for virulence genes.

Primary analyses will be conducted between residents with and without ASB at each time point. The primary outcome is UTI incidence during follow-up. In secondary analyses we will also take into account the low versus high presence of virulence genes of the *E. coli*.

**Discussion:**

The combination of high ASB prevalence and a reduced ability of frail older adults to express UTI-related S&S may lead to UTI misdiagnosis and inappropriate antibiotic use. To our knowledge, this is the first study to investigate the dynamics and role of bacteriuria in the elderly bladder and their potential protective effect on the development of UTI.

The study findings with comprehensive analysis of epidemiological, clinical and molecular data could set the fundamental base for future guidelines and studies, and contribute to improving prevention, diagnosis and treatment of UTI in frail older adults, in addition to contributing to antibiotic stewardship in NHs.

**Supplementary Information:**

The online version contains supplementary material available at 10.1186/s12879-024-09727-w.

## Background

Until recently, the presence of bacteria in urine (i.e., bacteriuria) of frail older adults, in combination with any of a large array of signs and symptoms (S&S), was considered pathogenic for urinary tract infections (UTIs). However, studies demonstrated that bacteriuria without any S&S related to UTI, such as dysuria and frequency (i.e., asymptomatic bacteriuria (ASB)) is present in up to 50% of nursing home (NH) residents. A causality between S&S and bacteriuria has not yet been found and the presence of bacteriuria as indicator for UTI has been debated [1]. Up until now UTIs are the most commonly diagnosed type of healthcare-associated infections in long-term care facilities such as NHs, and account for the majority of antibiotics prescribed [2–5]. As UTIs seem to be often diagnosed incorrectly, for instance solely based on the presence of ASB whether or not in combination with wrongly assigned or interpreted S&S, a substantial part of these antibiotic prescriptions are considered inappropriate varying from 32 to 73% [6–9].

This has led to a consensus statement from the American Medical Directors Association’s (AMDA) infection advisory subcommittee with two major adjustments in the management of UTI in NHs [1, 10]. First, UTI-related S&S need to be present for a UTI diagnosis. Hence, the presence of only non-related S&S, such as altered mental status, does not justify a UTI diagnosis. Second, urine testing for bacteriuria (e.g., urine dipstick or urine culture) should only be considered to rule out UTI diagnosis, not to confirm the diagnosis [11].

This paradigm shift, in which solely the presence of bacteriuria is no longer seen as an infection but as a possible colonization, poses new challenges for practice. NH residents with cognitive impairments often have difficulties to notice and communicate possible UTI-related S&S such as dysuria. The identification of S&S is further complicated by NH residents often being urinary incontinent and suffering from multiple comorbidities. Hence, there is a need for better diagnostic tools not solely based on S&S or the presence of bacteriuria [5, 12, 13].

The *Escherichia coli* (*E. coli*) is in general the most commonly found bacterium in urine cultures responsible for UTIs [14, 15]. This bacterium is part of the commensal intestinal microflora and very versatile with different variants responsible for diverse intestinal and extraintestinal diseases by the means of virulence factors that affect a wide range of cellular processes. The uropathogenic *E. coli* (UPEC) bacteria in particular are mostly responsible for UTIs.

The UPEC has acquired an array of virulence and antimicrobial resistance genes to colonize and attach to the urinary tract (e.g., fim genes), protect itself with biofilms (e.g., csg genes), produce toxins to inflict tissue damage (e.g., hly, cnf and sat genes) and be resistant against antibiotics commonly used to treat UTI (e.g., fos and blaCTX-M-genes). Depending on the presence and combinations of genes encoding different virulence factors, a UTI could be limited to a local cystitis or may manifest into a pyelonephritis [16–18]. Applying modern molecular techniques to distinguish high virulence bacteria causing UTI from low virulence bacteria has the potential to support physicians in diagnosis and treatment of UTI in the NH population.

Our hypothesis is that colonization of the bladder with low virulence bacteria (“good bacteria”) protects against the development of UTI with high virulence bacteria (“bad bacteria”), similar to the potential protection of commensal flora in the gastrointestinal tract [19–22]. If this is true, inappropriate use of antibiotics would eradicate the commensal “good bacteria”, which may have a deleterious effect on this defence mechanism of the elderly bladder. This hypothesis is supported by a study among young women with recurrent UTIs where the presence of ASB was associated with a lower risk of UTI, and in which it was shown that colonization of *Enterococcus faecalis* might be protective [23].

As mentioned in international guidelines on ASB, there is a need to evaluate potential biomarkers, such as leukocyturia (i.e., presence of leukocytes in the urine) for inflammatory response, to differentiate ASB and UTI in frail older adults [1]. A recent study has demonstrated that leukocyturia is commonly present in older women and concluded that current leukocyturia cutoffs are too low, promoting inappropriate UTI diagnosis [24]. However, in frail older adults, a correlation between the presence of an inflammatory response, S&S of a presumed UTI and an actual UTI has not been described to date. A common practice to evaluate the inflammatory response is by conducting a urinary dipstick test that, in addition to nitrite, demonstrates presence of leukocyte-esterase. Its usefulness to confirm UTI has however been debated given its sensitivity and specificity varying between 74–92% and 55–71% in the frail older adults [11].

Antibiotic use for incorrectly diagnosed UTI unnecessarily exposes patients to potential side effects and drug interactions, as well as to undertreatment if the true underlying cause of the UTI-related S&S remains untreated [4]. In addition, it contributes to the development of antimicrobial resistance, one of the biggest threats to global health [25]. Given the still unknown natural course of ASB in frail older adults, we aim to provide more insight into bacteriuria, leukocyturia and the genetic profile of *E. coli* in the elderly bladder in relation to the development of UTI in frail older adults. By doing so, the YELLOW RoUTIne study (*Insight in the dYnamics of bacteria in the ELderly bLadder: the way fOrWard to Urinary Tract Infection prevention, diagnosis and treatment)* may contribute to improved diagnosis and management of UTI in frail older adults.

## Methods

### Objective and hypothesis

The first objective of the YELLOW RoUTIne study is to describe the dynamics in the occurrence of bacteriuria and leukocyturia in urine samples of NH residents. This objective will be addressed by the following research questions:

A. What is the point-prevalence of ASB and leukocyturia in the study population at each time point, and what is the incidence of UTI?
B. Which bacteria are found in urine of residents with ASB or UTI and do these change over time in consecutively collected urine samples?
C. Are there differences and/or concordances between the ASB and UTI group concerning bacteria, patient characteristics, symptomatology and leukocyturia?

The second objective is to provide insight in virulence and antimicrobial resistance genes in *E. coli* identified in urine samples of NH residents, which will be addressed by the following research questions:

A. Which (set of) “relevant” virulence and antimicrobial resistance genes are identified in *E. coli* bacteria in NH residents with ASB and UTI, and can these be categorized respectively into “low” and “high virulence”?
B. How do virulence and antimicrobial resistance genes change over time in *E. coli* identified in consecutive urine samples from the same NH resident collected at the time of ASB or UTI?
C. Do NH residents with low prevalence of virulence genes in *E. coli* bacteriuria have reduced risk of subsequent UTI during follow-up?

The study’s main hypothesis will be tested by addressing the final research question (2C) in which we hypothesize that NH residents with “low virulence” *E. coli* ASB reduces the risk of a subsequent UTI.

### Study design and setting

**Fig. 1:**
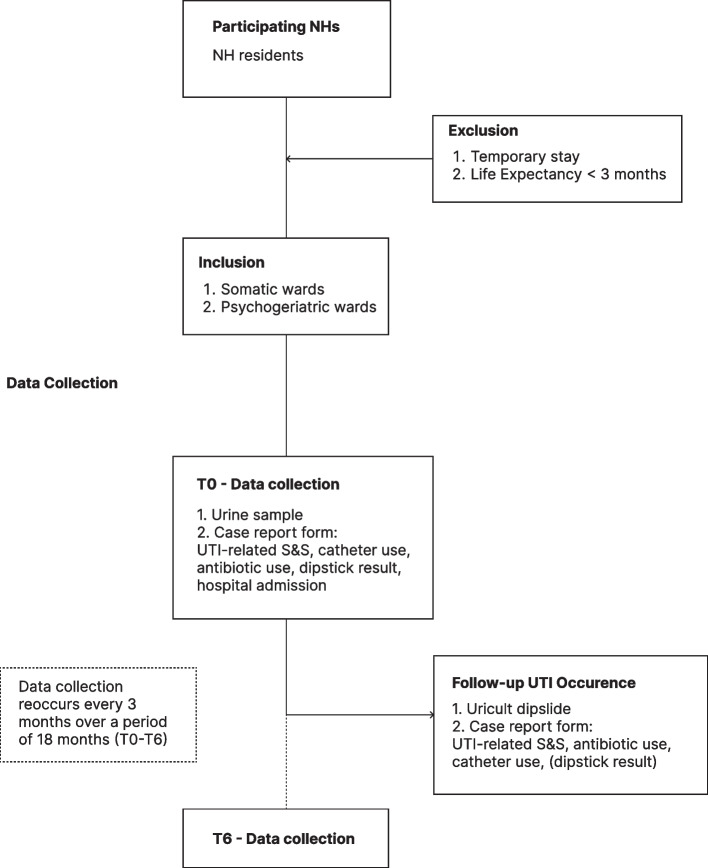
Study design and data collection

The YELLOW RoUTIne study is a prospective observational cohort study conducted in Dutch NHs. Data collection takes place between February 2024 and December 2025, and is planned to last 18 months in each participating NH [Fig. 1. Study design and data collection].

Medical care in Dutch NHs is primarily provided by ‘elderly care physicians’, physicians specialised in the care of frail older adults. These physicians are generally employed by the NH and have their main site of practice in the NH [26].

NHs were recruited through the Amsterdam educational institution for elderly care physicians (Gerion), the Amsterdam UMC University Network of Organisations for care for older adults (UNO Amsterdam), the University Network for the Care sector South Holland (UNC-ZH), Regional Cooperative Networks Antimicrobial Resistance (AMR zorgnetwerk) and the National Sentinel Surveillance Network of the Dutch National institute for Public Health and Environment [27–29].

### Recruitment of NH residents

NH residents are included in the study if they reside in long-stay somatic wards (i.e. residents with primarily physical impairments) or psychogeriatric wards (i.e. residents with primarily cognitive impairments). Residents with temporary stay, such as on geriatric rehabilitation wards, and residents with a life expectancy of less than three months are excluded.

All eligible NH residents are recruited by their caregivers and/or physicians of participating NHs. Written informed consent for study participation is obtained from either the resident or, in case of decision-making incapacity, their legal representative. The informed consent procedure is a continuous process during the first 9 months of the data collection, allowing new NH residents to participate for the remaining duration of the study.

## Data collection

### Urine samples

During the 18 month period of data collection, urine samples are collected from participating NH residents every 3 months (i.e., 7 time points in total per NH). This is done by their caregivers during standard routine of care. Urine samples are collected from spontaneously voided urine (in accordance with local procedures), an indwelling catheter (in accordance with local procedures), or urine-saturated incontinence material (see below). The research team further processes the samples and subsequently transport them to the study laboratory in accordance with “The Agreement concerning the International Carriage of Dangerous Goods by Road” (ADR) guideline [30].

In addition, whenever a physician suspects a UTI in between the time points, they are instructed to conduct a dipslide test (Uricult®, Aidian), a slide with agar to promote growth of possible uropathogens.

### Incontinence material

In order to prepare a sample for urinalysis, urine-saturated incontinence material worn for less than 12 h of NH residents that are unable to provide a voided urine sample, is processed on site by the research team at each time point (i.e., every 3 months) [Supplementary material 1, Standard Operating Procedure for incontinence material processing]. The procedure is based on earlier research on urine sample collection from incontinence materials [31].

In case of a UTI-suspicion in between the time points in incontinent NH residents, caregivers and physicians are instructed to press the dipslide onto the urine-saturated incontinence material for 10 seconds.

In both cases, incontinence material can only be used if no visible faecal contamination is present.

### Case report form

Research data collection is processed in the electronic clinical data management platform CastorEDC [32]. At each time point, any UTI-related S&S are gathered directly from the residents or their caregivers; remaining data are gathered from the electronic health record (EHR). These data include: baseline demographics (e.g., gender, age, type of residing ward), medical history (e.g., Charlson comorbidity index score [33]), renal diseases (recurrent UTIs, kidney stones, surgery), indwelling catheter use, incontinence (urinary/faecal), antibiotic use and hospital admission.

## Urinalysis

### Urine samples

All urine samples undergo dipstick and culture analyses. The urine dipstick test (Combur®, Roche) for nitrite and leukocyte-esterase is conducted on site by the study team at each time point.

For the urine samples collected upon UTI suspicion in between the time points, NH staff may conduct a urine dipstick test if this accords to their practice, in which case the research team collects the results from the EHR, but urine dipstick test performance is not requested for the study to prevent interference with usual care.

In the study laboratory the urine samples are automatically inoculated with the “Walk Away Specimen Processor” (WASP®, COPAN) onto the culture plates (blood agar, MacConkey and CHROMagar Orientation) while dipslides are manually inoculated onto blood agar and chromagar orientation in accordance to regular procedures. The culture plates are incubated for 18–24 h (37°C), aerobic conditions. An overnight incubation might be necessary for dipslides with no growth before manual inoculation.

Colony-forming units (CFU) is based on the number of colonies visible on the culture plate in accordance to regular procedures: 0 =  < 10^3^, 1–10 = 10^3^–10^4^, 10–100 = 10^4^–10^5^, > 100 =  > 10^5^. Identification of uropathogens takes place from CFU > 10^3^ for urine samples and CFU > 10^4^ for dipslides, with a limit of 3 uropathogens per culture [Table 1. List of uropathogens]. All cultured *E. coli* are stored at -80 degree Celsius and the remaining *non-E. coli* species (e.g., *Klebsiella* and *Proteus species*) are kept at -20 degree Celsius.

**Table 1:** List of uropathogens

*Actinotignum schaalii*
*Actinotignum sanquinis*
*Aerococcus urinae*
*Alloscardovia omnicolens*
*Coagulase-negative Staphylococci (CNS)*
*Enterobacterales*
*Enterococcus spp*
*Gardnerella spp*
*Yeasts (e.g., Candida albicans)*
*Gram negative cocci*
*Haemolytic streptococci*
*Lactobacillus delbrueckii*
*Non-fermenters (e.g., Pseudomonas aeruginosa)*
*Staphylococci spp (e.g., S. saprophyticus and S. aureus)*

### Cultured *E. coli*

Cultured *E. coli* undergo additional analyses. For antibiotic susceptibility testing a combination of different methods will be used depending on the cultured isolate, such as disk diffusion, E-test and the VITEK®-system (bioMérieux). Whole genome sequencing (WGS) will be conducted to determine the presence of virulence and antimicrobial resistance genes in *E. coli* [18]. We aim to perform WGS using Illumina or Nanopore sequencing technologies. However, due to current ongoing local and international developments, the exact WGS method has yet to be determined. Tools such as VirulenceFinder and ResFinder may also be used to determine virulence gene profile [34, 35].

### Low & high virulence definition

To define the difference between low and high virulence we will conduct WGS on a selection of the cultured *E. coli.* The different identified (virulence) genes and combination of (virulence) genes will be analyzed with a multiple regression analyses and/or a principle component analysis. The goal is to identify (sets of) virulence genes within the genome of the cultured *E. coli* from NH residents with UTI which could be considered as “high virulence”, while the absence of these (sets of) virulence genes could be used to define “low virulence”. The exact combination and threshold for “high virulence” has yet to be defined by a workgroup consisting of medical microbiologists and infectiologists based on analyses of the study data and review of the literature.

### UTI & ASB definition

The clinical UTI definition used in the current study is in line with current (national) UTI guidelines in NH residents [36, 37]. The resident needs to have one or more, or a very bothersome, of the following recent onset UTI-related S&S: dysuria, urgency, frequency, incontinence, (visible) urethral purulence, whether or not combined with any systemic signs of infection (fever, rigors/shaking chills and clear-cut delirium) or costovertebral angle pain/tenderness and/or suprapubic pain. Alternatively, a UTI diagnosis may be based on the presence of costovertebral angle pain/tenderness in combination with any systemic signs with no other infectious focus. In residents with indwelling catheter, a UTI diagnosis is based on the presence of systemic signs with no other infectious focus.

ASB is defined as the absence of the abovementioned criteria in combination with a positive urine culture for any uropathogens.

## Statistical analysis

### Sample size calculation

The sample size was calculated for testing the main study hypothesis (research question 2C). As the definition of “low” versus “high virulence” *E. coli* has to be determined during study conduct, the sample size calculation is based on the hypothesis that there is a difference in UTI incidence after 1,5 years between NH residents with and without ASB at baseline. Data from the national sentinel surveillance network of the Dutch National institute for Public Health and Environment show 1.751 registered UTI in an average number of 2.350 NH beds over a 1,5 year period. Based on previous research findings, we assume that 30% of these UTI registrations are recurrent UTIs, which are excluded for the current sample size calculation [6]. This leaves 1.226 non-recurrent UTI registrations per 2.350 NH beds. For the main analysis, we focus on UTI in residents without an indwelling urinary catheter. Based on previous study findings, we anticipate 15% of the UTI episodes to occur in residents with an indwelling urinary catheter [6, 38]. Correction for this leaves 1.042 non-recurrent UTI registrations per 2.350 NH beds. Thus, the chance of UTI in 1,5 years is on average 44%.

We consider an absolute difference in incidence between residents with and without ASB of 15% clinically relevant. In other words, the chance of UTI in 1,5 years is 36,5% in residents with ASB at baseline and 51,5% residents without ASB at baseline (relative reduction of 15/51,5 = 29%). Presuming a power of 0.80 and alpha of 0.05, we need 182 NH residents per group (364 in total) [39].

Taking into account a loss-to-follow-up of 50% results in a required total number of 728 residents. Furthermore, taking into account that 50% of the approached NH residents provides informed consent, we need to approach 1.456 NH residents.

The outcome measure in this calculation is defined as the development of UTI as per national guideline during follow-up. However, due to repeated measures, multiple UTIs can occur in one resident which potentially results in a larger power.

### Outcomes

The primary study outcome is the incidence of clinical UTI during follow-up. Secondary study outcomes include the course of UTI such as presence of any (non-)UTI-related S&S, antibiotic use, hospitalization and mortality; point-prevalence of ASB, UTI and leukocyturia; and presence of “low/high virulence” *E. coli*.

### Data analysis

By analysing and comparing the composition of the consecutively collected urine samples, it will be possible to describe the course and underlying causes of bacteriuria in NH residents (objective 1). Descriptive analyses will be applied for research question 1A and 1B and logistic mixed model analysis with a three-level structure (repeated measurements are clustered within NH residents, and NH residents are clustered within NHs) will be used to investigate any possible associations between the ASB and UTI group concerning bacteria, patient characteristics, symptomatology and leukocyturia (research question 1C). We mainly focus on residents without an indwelling urinary catheter and with urine collected during spontaneously voiding moments (CAD-/voiding +). A separate analysis is conducted on residents with urine collected from incontinence material (CAD-/voiding-) and residents with an indwelling urinary catheter (CAD + /voiding-).

For objective 2, the presence (or absence) of virulence and resistance genes will be descriptively analysed for the *E. coli* within the ASB and UTI group, and within one resident during follow-up. WGS results will also be descriptively analysed and resistance gene results (genotypic) will be compared with antimicrobial susceptibility test results (phenotypic) (research questions 2A & 2B). For research question 2C, the association between the time-varying exposure (i.e. prevalence of ASB at a certain time point) and the time-varying outcome (UTI) in the following three months, will also be analysed with a three-level logistic mixed model analysis. In a secondary analysis, this association will be studied using a three-group exposure: no ASB, “low virulence” ASB and “high virulence” ASB.

## Discussion

This protocol paper describes the design of a prospective observational cohort study that aims to provide more insight in the presence of bacteriuria, leukocyturia and the genetic profile of *E. coli* in the bladder of NH residents, and in its relation to UTI.

### Reflection on study design

First of all, the long follow-up period combined with the limited life expectancy of the study population complicates certain aspects of this study design, such as the sample size calculation. The Dutch National Health Care institute reported that approximately 14% of the residents decease within 3 months after admission and only 45% of the residents have a length of stay over 2 years on reference date 2018 [40]. Considering this, we have taken into account a loss-to-follow-up rate of 50% in the sample size calculation and extended the inclusion period up till halfway the data collection period to reach the intended sample size. Whereas patients with cognitive impairments are often excluded from study participation, we explicitly included this group as they represent a crucial part of the NH population and a group in which UTI diagnosis can be particularly challenging [41, 42].

Secondly, the definition of UTI has been the focus of recent debate. Although studies have evaluated various antibiotic stewardship interventions for UTI in NHs, physicians still encounter difficulties in implementing existing guidelines to improve appropriate UTI diagnosis [38, 43]. To address possible “mis-diagnosis” of UTI in our study, we will conduct subgroup analyses distinguishing “physician-reported” UTI from “guideline-adherent” UTI [10].

Another point of discussion is the method of urine sample collection [44, 45]. While clean mid-stream urine is the preferred collection method to minimize contamination, this is not always feasible in frail older adults. Urinary incontinence is common in this population, with a prevalence ranging from 43 to 77% [5, 12, 13]. Our use of incontinence material for urinalysis is based on a recent laboratory study showing promising results that are comparable with standard practice [31].

Finally, for logistic reasons we decided to use dipslides as it allows us to culture and collect any uropathogens whenever a UTI suspicion occurs (i.e., also during out-of-hours). Similar studies have also used the dipslide for laboratory culture and stated that this did not affect the reliability of the culture results [46, 47].

### Possible future implications

Ultimately, with the YELLOW RoUTIne study, we aim to contribute to the development of hands-on and easily applicable urinalysis results for the management of UTI in frail older adults, in turn leading to more appropriate antibiotic prescribing.

In the future a physician may not only obtain a urinalysis report stating “*E. coli* ≥ *10*^*5*^* CFU/mL*” but also an extended report describing the presence of certain virulence genes and its clinical implications with a conclusion and treatment advice, which the treating physician can use in combination with the presence or absence of certain S&S of the patient. For example: “*The detected bacteria is considered a high virulence E. coli and associated with a UTI, with presence of the antimicrobial resistance gene to Fosfomycin. Treatment is advised unless the patient has no clinical relevant symptoms.”*

Though our primary focus is on *E. coli*, the common cause for UTIs, the remaining bacteria (e.g., *Proteus*, *Enterococcus* and *Klebsiella species*) are still preserved [15]. Similar tests may be conducted for these species in future studies.

In conclusion, the study findings with comprehensive analysis of epidemiological, clinical and molecular data could set the fundamental base for future guidelines and studies, and contribute to improving prevention, diagnosis and treatment of UTI in frail older adults, in addition to contributing to antibiotic stewardship in NHs.

### Supplementary Information

Supplementary Material 1

## Data Availability

The datasets generated during the study will be deposited after publication of study results. The datasets involved will be pseudonymised and can be accessed under restrictions.

## Abbreviations

ADR: The Agreement concerning the International Carriage of Dangerous Goods by Road
AMDA: American Medical Directors Association
AMR zorgnetwerk: Regional Cooperative Networks Antimicrobial Resistance
ASB: Asymptomatic bacteriuria
CFU: Colony forming units
CRF: Case report form
E. coli: Escherichia coli
NH: Nursing home
S&S: Signs & symptoms
UNC-ZH: The University Network for the Care sector South Holland
UNO Amsterdam: University Network of Organizations for care for older adults
UPEC: Uropathogenic Escherichia coli
UTI: Urinary tract infection
WASP: Walk Away Specimen Processor
WGS: Whole genome sequencing
