# Supplementary material for: YELLOW RoUTIne prospective cohort study protocol: insight in the dynamics of bacteria in the elderly bladder
Source: BMC Infect Dis. 2024 Aug 30;24:890. doi: 10.1186/s12879-024-09727-w (PMC11363575; doi:10.1186/s12879-024-09727-w)
Supplement: Supplementary file 1 — Supplementary Material 1 [file 12879_2024_9727_MOESM1_ESM.pdf]

Supplementary material 1

Standard operating procedure: Incontinence material processing

Background

Urine sample collection is often difficult for NH residents due to cognitive impairments and/or urinary incontinence. Caregivers are instructed to collect urine-saturated incontinence material (without fecal contamination) at each time point if spontaneously voided urine is not possible.

Materials

- Urine container
- Physiological saline (0.9% NaCl solution)
- Scissors
- Pincers
- Single-use disposable wooden spatulas (14 x 0.5cm)
- Alcohol podior 70%

Procedure

- Label the urine container with the appropriate participant sticker.
- Add 9 mL physiological saline to the urine container.
- Open the incontinence material with a scissor by cutting through the first layer revealing the urine-saturated pulp-fiber layer underneath.
- Remove approximately 2-3 cm^2 urine-saturated pulp-fiber with a pincer and add this to the urine container containing 9mL physiological saline.
- Stir for 10 seconds.
- Push the pulp-fiber with a single-use disposable wooden spatula against the edge of the container to release the urine-absorbed physiological saline back into the container.
- Remove the pulp-fiber along with the wooden spatula and close the urine container.
- Disinfect the scissor and pincer with 70% alcohol.
